# Individual variation and seasonality drive bird feeder use during winter in a Mediterranean climate

**DOI:** 10.1002/ece3.4902

**Published:** 2019-02-14

**Authors:** Janel L. Lajoie, Lisa M. Ganio, James W. Rivers

**Affiliations:** ^1^ Department of Forest Ecosystems and Society Oregon State University Corvallis Oregon

**Keywords:** bird feeding, Black‐capped Chickadee, PIT‐tagging, *Poecile atricapillus*, RFID, supplemental feeding

## Abstract

Purposeful provisioning of food to wild animals is a widespread and growing activity that has the potential to impact populations and communities. Nevertheless, studies assessing use of recreational feeders by free‐living birds during winter are surprisingly rare and largely limited to regions with continental climates characterized by freezing temperatures and snow cover. In contrast, there is little information available regarding bird use of feeders within warmer climates during winter, despite widespread recreational feeding in these areas. In this study, we quantified visitation patterns to bird feeders in a Mediterranean climate to evaluate the relationship between feeder use and several environmental variables known to influence supplemental feeder use in continental climates. We established a network of bird feeders in Corvallis, Oregon, USA, that were filled with black oil sunflower (*Helianthus annuus*) seeds and equipped with radio frequency identification (RFID) data loggers that recorded >315,000 visits by 70 individual Black‐capped Chickadees (*Poecile atricapillus*) across a 5‐month period (October 2016–March 2017). We found extensive variation in feeder use, with individuals averaging 1–406 feeder visits/day and using 1–9 of the 21 feeders that were available; individual variability was largely consistent during the course of our study. At the population level, we found that feeder use decreased from the start of our study, and this decline continued through the period when foraging was most limited by daylight, including the winter solstice. In contrast to theoretical predictions and empirical work in continental climates, we found that weather variables did not drive feeder use and that feeder visits peaked at mid‐day and gradually decreased until sunset. Our study indicates that individual‐level differences combined with seasonality to drive feeder use patterns, and we conclude that use of supplemental feeders during winter in Mediterranean climates appears to differ notably from feeder use in continental climates.

## 1 INTRODUCTION

Wild animals can be provided with anthropogenic food incidentally, through waste generated by population centers, food production facilities, or agricultural production, or purposefully through direct provisioning of food resources (reviewed by Oro, Genovart, Tavecchia, Fowler, & Martínez‐Abraín, 2013). In many parts of the world, purposeful provisioning of food to wild animals is a widespread and growing activity that has given rise to a global wildlife feeding industry (Galbraith et al., 2014; Jones & Reynolds, 2008). In the United States alone, for example, more than 53 million people report feeding wildlife around their homes with the great majority (95%) providing food for wild birds. This results in approximately US $5 billion per year spent on bird seed and bird feeding accessories, which represents a doubling in just the last two decades (USFWS, 1991, 2011). More broadly, up to 75% of households in industrialized nations are thought to regularly feed wild animals (Cowie & Hinsley, 1988; Davies, Fuller, Dallimer, Loram, & Gaston, 2012; Galbraith et al., 2014), making food supplementation of wildlife, particularly birds, a widespread and global phenomenon.

Given the scale at which it occurs, purposeful feeding of wild animals has the potential for widespread consequences for natural populations. It is well known that food resources underlie many demographic processes (Fischer & Miller, 2015; Robb, McDonald, Chamberlain, & Bearhop, 2008), and that populations can increase when natural diets are supplemented with regular, predictable anthropogenic food sources (Oro et al., 2013). Empirical studies have found that supplemental feeding of wild bird populations can alter foraging effort, territoriality, and the phenology of reproductive behaviors (Robb, McDonald, Chamberlain, Reynolds et al., 2008), and supplemental food has also been linked to increases in individual survival (Brittingham & Temple, 1988; Jansson, Ekman, & Bromssen, 1981; Orell, 1989) and improved condition (Desrochers & Turcotte, 2008; Grubb & Cimprich, 1990; Wilcoxen et al., 2015). Conversely, supplemental feeding can exert negative effects on populations that include increased disease transmission rates (Adelman, Moyers, Farine, & Hawley, 2015; Becker, Streicker, & Altizer, 2015), promotion of feeder dependency (Robb, McDonald, Chamberlain, & Bearhop, 2008), and a reduction in health via provisioning of nutrient‐poor foods (Jones & Reynolds, 2008). Furthermore, supplemental feeding has also been linked to evolutionary changes in morphology (Bosse et al., 2017; Rolshausen, Segelbacher, Hobson, & Schaefer, 2009) and seasonal migration patterns (Plummer, Siriwardena, Conway, Risely, & Tomas, 2015), highlighting how intentionally provisioned food can lead to pronounced evolutionary changes in wild populations. Therefore, it is clear that intentional feeding of wildlife has the potential to influence wild populations in myriad ways, with both positive and negative consequences.

Despite the potential for intentional feeding to impact demographic and evolutionary processes, investigations that assess use of supplemental feeders by songbirds—the group most commonly targeted for intentional feeding—are surprisingly rare. For those studies that have quantified use of supplemental food, the great majority have been conducted in regions with continental climates that are characterized by cold winters with extensive snow cover and temperatures below freezing (Bonter, Zuckerberg, Sedgwick, & Hochachka, 2013; Cooper & Sonsthagen, 2007; Kessel, 1976; Lima, 1985; Wilson, 2001). Several studies have found that feeder use by songbirds in midwinter, when ambient temperatures reach seasonal lows and foraging time is constrained by short photoperiods, generally increased throughout the day until sharply declining as sunset approached and individuals moved to roost sites (Bonter et al., 2013; Monus & Barta, 2016). Feeder use by songbirds in continental climates has also been found to increase with decreasing ambient temperature (Bonter et al., 2013; Chaplin, 1976; Zuckerberg et al., 2011), and decrease with increasing wind speed (Kessel, 1976; Kubota & Nakamura, 2000), with both patterns thought to be driven by the energetic needs of birds in winter (Bednekoff & Houston, 1994; McNamara, Houston, & Lima, 1994). Although these findings have help establish our understanding of how wild birds make use of supplemental food, it is important to note that recreational bird feeding is not restricted to regions with continental climates but is instead practiced over a much broader area. For example, bird feeding is popular in many areas of the United States that are snow‐free and experience milder, nonfreezing temperatures during the winter (Horn & Johansen, 2013; Horn, Johansen, & Wilcoxen, 2014; https://feederwatch.org/). Given the strong influence of local environmental conditions on feeder use demonstrated by previous investigations, particularly weather (Bonter et al., 2013; Grubb, 1978; Kessel, 1976; Tryjanowski et al., 2015), the findings from studies conducted in continental climates may generalize poorly to regions with warmer climates. Therefore, new studies are needed that evaluate recreational feeder use under different environmental conditions experienced by songbirds during the winter season.

In this study, our goal was to quantify patterns of supplemental feeder use by wild, free‐ranging Black‐capped Chickadees (*Poecile atricapillus*, hereafter chickadee) during winter in a Mediterranean climate. Such climates are characterized by mild, rainy winters with no regular snow cover and temperatures above freezing (Kottek, Grieser, Beck, Rudolf, & Rubel, 2006) and therefore offer a strong contrast to the winter conditions typical of continental climates. In particular, evaluating supplemental feeder use in Mediterranean climates allows for winter conditions in which to test theoretical predictions that stem from the starvation–predation trade‐off. Small birds, such as the chickadee we studied (10–14 g, Foote, Mennill, Ratcliffe, & Smith, 2010), experience energetically challenging conditions that require them to maintain adequate energetic reserves to survive long, cold nights in winter, yet carrying additional reserves bears a cost because greater reserves can reduce maneuverability when escaping from predators (Bednekoff & Houston, 1994; Lima, 1986; McNamara et al., 1994). This means that individuals must maintain energetic reserves that prevent starvation, but they must also balance the risk of starvation against the risk of being captured by predators. When both conditions are important for winter survival, the daily foraging pattern is expected to be bimodal, with greater foraging activity early in the day (to recover reserves used overnight) and late in the day prior to roosting (to build reserves for the subsequent night); lower levels of feeding are expected during the remaining daylight hours (Bednekoff & Houston, 1994; McNamara et al., 1994). To date, empirical tests of this idea have been largely intractable because obtaining extensive, fine‐scale foraging records of individuals has been impossible. However, recent advances in animal tagging, namely the use of uniquely numbered passive integrated transponder (PIT) tags coupled with bird feeders equipped with radio frequency identification (RFID) data loggers that record visits of individual birds, have allowed researchers to quantify individual feeder use to a degree that was not possible in the past (Brittingham & Temple, 1992; Van Buskirk & Smith, 1989). These new techniques provide an especially valuable opportunity to evaluate relationships between feeder use and local environmental conditions throughout winter, including testing theoretical predictions regarding starvation–predation trade‐offs in wild, free‐ranging birds (e.g., Bonter et al., 2013; Pitera, Branch, Bridge, & Pravosudov, 2018).

In this study, we attached PIT tags to individual chickadees and monitored their use of bird feeders equipped RFID data loggers to assess how feeder use varied in three distinct ways. First, we quantified the extent to which individuals differed in feeder use across the winter season, as how often individuals make use of supplemental food likely reflects individual variation in behavior and physiology. Differences in behavioral and physiological traits of individuals are widespread in nature (Biro & Stamps, 2010; Careau & Garland, 2012; Careau, Thomas, Humphries, & Reale, 2008; Koolhaas et al., 1999; Sih, Cote, Evans, Fogarty, & Pruitt, 2012) and can drive population‐level changes (Wolf & Weissing, 2012), yet we have only a limited understanding of individual‐level use of supplemental feeders during winter when food resources and foraging time are expected to be most limiting. Second, we quantified how patterns of feeder use varied temporally on both short (i.e., hourly) and long (i.e., seasonal) timescales. Following theoretical work, we predicted that feeder use would be bimodal with visitation peaks concentrated early in the morning and late in the day, with reduced feeding rates throughout the rest of daylight hours (Bednekoff & Houston, 1994; McNamara et al., 1994). We also predicted that individuals would increase feeder use as photoperiod decreased until the point of the winter solstice (i.e., shortest day of the year with respect to light), and would then decrease as day length increased (Bonter et al., 2013). Third, we examined whether several environmental variables known to influence feeder use in colder climates were linked to feeder use in our study system. We examined minimum daily ambient temperature because birds require more food at lower temperatures to maintain homeostasis (Grossman & West, 1977; Kessel, 1976; Sharbaugh, 2001) and because feeder use generally increases as ambient temperature decreases (Bonter et al., 2013); here, we predicted that birds would increase feeder use as daily minimum ambient temperatures decreased (Bonter et al., 2013). Chickadees are known to seek shelter and delay their first appearance to feeders under windy conditions in some climates (Kessel, 1976), so we also quantified the effects of maximum daily wind speeds on feeder use, predicting that individuals would decrease feeder use within increasing wind speeds (Grubb, 1978; Kessel, 1976). Finally, we assessed feeder use relative to precipitation, predicting that birds would decrease use of feeders as precipitation increased following previous work (Zuckerberg et al., 2011).

## 2 MATERIALS AND METHODS

### 2.1 Study area and focal species

We established 21 bird feeders in September 2016 on lands owned by Oregon State University near the Corvallis, Oregon, USA campus (44.6°N, 123.3°W, 90 masl). We placed feeders at 200‐m intervals along the Oak Creek riparian area such that the two most distant feeders were 3.2 km away from each other; this distance is within the range of what an individual chickadee could theoretically cover in the course of our study period based on previous work with homing experiments conducted during winter (Odum, 1941) and studies of winter flock space use (Desrochers & Hannon, 1989; see Discussion). In two instances, we were forced to place feeders approximately 400 m apart because of site access restrictions. We installed each feeder within 60 m of a continuously flowing creek in habitat that was dominated by Oregon white oak (*Quercus garryanna*), Himalayan blackberry (*Rubus discolor*), bigleaf maple (*Acer macrophyllum*), poplar (*Populus *spp.), and willow (*Salix *spp.) and contained a mixture of native shrubs, grasses, and forbs. We selected feeder locations that were a minimum of 50 m from buildings, and although it is possible that our tagged birds could have accessed feeders other than those established for our study, we found that there was no evidence that use of individual feeders varied relative to their distance to the nearest building (*F*_1,19_ = 0.05, *p* = 0.82, *R* = 0.05; Supporting Information Figure S1). Therefore, we did not consider distance to buildings further in any of our analyses.

We selected the chickadee for this study because this species (a) is a small‐bodied songbird (10–14 g) with high energetic needs during the nonbreeding season (Foote et al., 2010; Smith, 1991), (b) is a year‐round resident that occurs in temporally stable flocks throughout the winter, at least in continental climates (Desrochers & Hannon, 1989; Smith, 1991), and (c) is one of the most common visitors of recreational bird feeders (Brittingham & Temple, 1989; Horn et al., 2014; Smith, 1991). In addition, chickadees typically remove a single sunflower seed from feeders during each visit before moving away from the feeder to consume or cache the seed (Ficken, Weise, & Popp, 1990; Foote et al., 2010), making the number of feeding visits a close proxy of the number of seeds obtained when estimating the amount of food obtained by individuals from feeders (Latimer, Cooper, Karasov, & Zuckerberg, 2018).

### 2.2 Chickadee PIT‐tagging and RFID‐equipped feeders

We captured chickadees from late October to early January in two consecutive winters (2015–2016, 2016–2017) from 08:00 to 17:00 PST on fair days (i.e., clear sky to light drizzle, wind speed <10 km/hr) using a combination of mist‐nets and traps that were affixed to feeders. We PIT‐tagged a total of 174 chickadees that were available for this study; 86 birds were captured during pilot work undertaken during October 2015–January 2016, and 88 birds were captured from October 2016–January 2017. We fitted each captured individual with a unique combination of legs bands that consisted of a USGS aluminum leg band, a colored plastic leg band, and a colored band that contained a uniquely numbered PIT tag (IB Technology, Aylesbury, UK). At initial capture, we also measured body mass and right tarsus length to assess variation in size among individuals.

**Figure 1:**
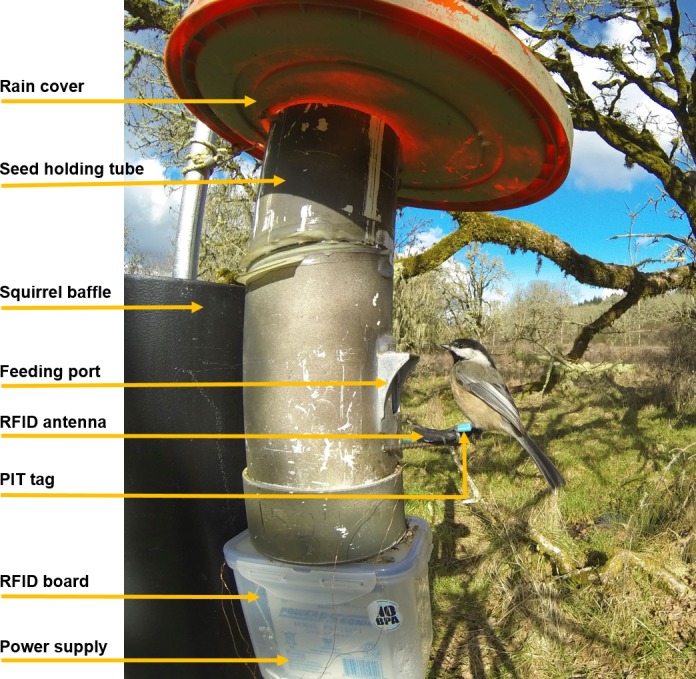
Example of a supplemental feeder used in this study to quantify feeder use by Black‐capped Chickadees in a Mediterranean climate in Corvallis, Oregon, USA. The light blue PIT tag is evident on the chickadee's left leg, but the aluminum leg band and colored plastic leg band on the bird's right leg is out of sight in this image. Note that the wire mesh trap attached to the feeder for capturing birds has been removed in this photograph for clarity

Each of our supplemental feeders consisted of a customized seed tube that had one feeding port at its base so that only a single chickadee could visit the feeder at a single time (Figure 1). We hung each feeder approximately 2.5 m off the ground from a metal pole that was fitted with a metal baffle to deter use of feeders by the western gray squirrel (*Sciurus griseus*) and the Douglas squirrel (*Tamiasciurus douglasii*). We also fitted feeders with a 10 × 15 cm wire mesh trap attached around the feeding portal to capture individual chickadees for tagging. Each feeder was capable of holding approximately 3 kg of unhulled black oil sunflower (*Helianthus annuus*) seeds, and we provided food ad libitum in all feeders throughout the course of the study. On the base of each feeder, we attached a small waterproof box that housed a 12‐volt battery for power and an RFID data logger (Bonter & Bridge, 2011), and we programmed the data logger to record all visits that occurred between 1 hr before sunrise and 1 hr after sunset. We affixed an antenna with a ~5‐cm detection range to the perch of the feeding port on each feeder that recorded feeder use by PIT‐tagged birds. Specifically, for each visit by a PIT‐tagged chickadee the unique bird identification number, unique feeder identification number, date, and time of day (to the nearest second) were recorded by the data logger, allowing us to get fine‐scale temporal data on feeder visits by all tagged individuals across the period of our study (i.e., October 23, 2016–March 31, 2017). Chickadees typically make short‐duration visits to bird feeders to obtain a sunflower seed before flying away to consume or cache the seed; therefore, we programmed RFID data loggers to scan for PIT tags once every 0.5 s which was followed by a 0.5‐s pause; this prevented us from missing especially short visits of tagged chickadees. Previous work has found that chickadees typically take >10 s to handle sunflower seeds obtained from feeders (Lima, 1985), which we confirmed in our study (Lajoie, 2018). Therefore, we considered a unique feeder visit to be one that was separated from other visits by at least 10 s to minimize the likelihood that individuals were double‐counted during a single feeder visit.

### 2.3 Vegetation cover surrounding feeders

Feeder use by birds may be influenced by nearby vegetation used to hide from predators (Cowie & Simons, 1991; Lima, 1985; Ya‐Fu, Yen‐Min, & Bollinger, 2005), so we quantified vegetative cover surrounding feeders as a measure of escape cover. To do this, we established a 20‐m radius circle that was centered at each feeder, and within each circle, we visually estimated the proportion that was covered by vegetation in the tree layer (>3 m above the ground), shrub layer (0.5–3.0 m), and herbaceous layer (<0.5 m). When multiple vegetation layers occurred, we measured vegetation closest to the ground first and then calculated the percentage for the next higher layer by subtracting the lower layer's measurement from the percent of ground surface covered by this next vertical layer. We used these data to identify differential use of feeders due to the presence of escape cover; however, we found no relationship between the extent of feeder use and vegetation cover (*F*_1,19_ = 2.64, *p = *0.12, *R* = 0.35). Thus, we concluded that feeder use was not linked to escape cover immediately surrounding feeders and therefore did not consider vegetative cover in any subsequent analyses.

### 2.4 Weather data

We obtained weather data recorded at the Corvallis Municipal Airport in Corvallis, OR (44°33′N, 123°15′W) by the National Oceanic and Atmospheric Administration (NCEI, 2017) for each day during which we quantified chickadee feeder use. The weather station was located within 9 km of all feeders, and we assumed it to be representative of local conditions experienced at those feeder locations. We selected variables known to influence feeder use from prior studies which included: minimum daily ambient temperature (°C, hereafter *T*_min_), average daily wind speed (km/hr, hereafter wind speed), and total daily precipitation (mm, hereafter precipitation). In addition, we obtained sunrise and sunset times for the location of the weather station from the Astronomical Applications Department of the U.S. Naval Observatory (Department of Defense, 2011) and calculated photoperiod length by subtracting sunrise time from sunset time for each day during which we quantified chickadee feeder use (hereafter photoperiod). For analysis, we binned photoperiod into 1‐hr blocks across the daylight period. When a feeder visit occurred in the hour before sunrise, we assigned it to 0 with respect to the hour from sunrise; when a feeder visit occurred ≥1 hr from sunrise, we considered the hour since sunrise to be 1, with the same approach taken for successive hour increments.

### 2.5 Energetics of black oil sunflower seeds

To determine the amount of potential energy that chickadees acquired from feeders, we had extraction analysis performed by the Linus Pauling Institute at Oregon State University on a haphazardly selected sample (*n* = 20) of hulled black oil sunflower seeds taken from the pool of seeds used in feeders in our study. Initially, the sample was separated into two equal groups (i.e., 10 seeds) and analysis took place on a 0.2 g sample of dried and ground sunflower seeds from each group. Next, samples were combined with 4 ml 50% MeOH:H_2_O with 1% HCl, and 450 µl 17:1 of FFA IS2, an internal standard added to all samples to aid with quantitation. After 10 min of vortexing and sonication, 4 ml of chloroform was added, followed by a second 10‐min vortex and sonicate session, and then centrifuged 10 min at 1,000 G. After saving the lower layer, the upper layer was re‐extracted in the same manner as above. The lower layer was then combined with the re‐extracted upper layer, and a 3 ml 0.88% KCl solution was added, followed by 5 min of vortexing and sonication, then centrifuged 10 min at 2,000 G to achieve phase separation. The upper layer was removed and discarded, chloroform:MeOH 2:1 was added, the sample was dried under N_2_, and then the extracted lipids were weighed. The lipid extraction yielded an average of 0.26 kcal of fat per seed.

To quantify how many black oil sunflower seeds a chickadee would require to fulfill its daily energy requirements, we assumed that chickadees required 65.5 kJ of energy/day (Karasov, Brittingham, & Temple, 1992), that the rate of metabolizable energy from a sunflower seed was 0.865 (Wu, 2018), that lipids were the primary energy source individuals obtained from sunflower seeds, and that individuals only consumed sunflower seeds to obtain energy. Using our empirical estimates of fat content, we determined that an individual chickadee required approximately 70 sunflower seeds/day to cover its entire daily energy expenditure (DEE). Given that chickadees do not subsist on seeds alone in the winter (Smith, 1991) and that previous estimates of energy use were taken from locations with lower ambient temperatures than those of our study site (Karasov et al., 1992), our estimate of 70 seeds/day is likely to be an upper limit of what an individual bird in our study population would need to consume in a single day during the course of our study.

### 2.6 Statistical analysis

To address the relationship between feeder use and our focal variables, we computed two response variables for each PIT‐tagged bird. For the first response variable, we calculated the number of visits made to all feeders on each day that an individual visited at least one feeder during the study period (October 23, 2016–March 31, 2017), hereafter termed “total daily visits.” We used this metric to assess how feeder use varied between individual chickadees and quantify the extent to which it was linked to weather variables. To quantify individual‐level variation in feeder use, we summarized the distribution of total daily visits for each bird and the number of feeders visited across the season for each bird, using variance estimates of random effects to quantify the ratio of among‐bird variation to within‐bird variation from our statistical model.

To assess how weather variables were linked to feeder use, we undertook a modeling approach to assess how mean total daily visits were associated with *T*_min_, wind speed, precipitation, and photoperiod. Exploratory analysis of data prior to modeling found that the general pattern of response was similar among individual birds; however, the magnitude of the response exhibited large variation, so we included a random effect for each bird in all models. Because total daily visits were measured on multiple days for each bird, we graphically assessed the temporal autocorrelation function of residuals in each of our single‐variable models and found that a first‐order autoregressive model had the best fit for the temporal autocorrelation among total daily visits. We found that mean total daily visits were best modeled with a negative binomial distribution allowing for overdispersion. In addition, our initial graphical assessment of total daily visits relative to weather variables found that mean total daily visits varied differently before and after the winter solstice. Thus, we investigated whether effects of environmental variables differed between the pre‐solstice period when photoperiod was decreasing, and the postsolstice period when photoperiod was increasing. We fit generalized linear mixed models (GLMMs) using the glmmTMB package (Magnusson et al., 2017) in R version 3.4.0 (R Core Team, 2017), and we used Akaike's Information Criterion (AIC) statistics to rank the degree of support for each regression model. We calculated AIC weights and evidence ratios for our models of mean total daily visits (Burnham, Anderson, & Huyvaert, 2011).

We regressed total daily visits on *T*_min_, wind speed, precipitation, photoperiod, and an indicator variable for the postsolstice period. In an effort to evaluate a small but meaningful set of potential models (Burnham et al., 2011), we fit three models for each weather variable. We fit a model that contained only the weather variable, a second model that contained the weather variable and the interaction of the weather variable and the postsolstice indicator, and a third model that contained the weather variable, the interaction of the weather variable and our postsolstice indicator, and all remaining weather variables. We also fit a null model that contained no weather variables.

We used a two‐step process to evaluate the influence of weather variables. First, we compared the first and second model to the null model to evaluate the association between the weather variable and the response variable, and whether the slope of the association differed before and after the solstice. We concluded that the relationship differed before and after the solstice if the AIC for the 2nd model was two or more units less than the AIC for the first model and the null model. If the AIC for the first model was two or more units less than the AIC for the second model and for the null model, we concluded that the relationship was similar before and after the solstice. We concluded that there was no support for the weather variable if the AIC for the null model was two or more units less than the AIC for the first and second models, or if the AIC for the null model was within 2 AIC units of the model with the lowest AIC. In step two, we compared the third model to the model with the smallest AIC in the first step to evaluate whether the weather variables, in combination with each other, were associated with the response variable. We concluded that weather variables were needed in combination if the AIC from model three was 2 or more AIC units less than the AIC from the best‐supported model in step 1.

For our second response variable, we summed the number of visits made to all feeders during each hour since sunrise for each day from November 9, 2016 through January 31, 2017, hereafter termed “total hourly visits.” We restricted this analysis to this period because day length during this period varied minimally (8.8–9.8 hr) and resulted in the shortest foraging days for chickadees during our study. As above, we used a Poisson GLMM with the glmmTMB package (Magnusson et al., 2017) in R version 3.4.0 (R Core Team, 2017) to quantify the relationship between the mean total hourly visits and hour since sunrise. We included the number of days each bird visited any feeder within each hour since sunrise as an offset in the model to adjust for the varying number of days an individual bird visited during each hour of the day. We graphically assessed the autocorrelation function of residuals and used AIC statistics to identify the best‐supported autocorrelation structure. We found the relationship between total hourly visits and time since sunrise was concave but asymmetrical, suggesting that a linear or quadratic relationship between mean total hourly visits and time since sunrise would have been a poor fit, and a cubic relationship with multiple changes of slope did not occur. Therefore, we treated hour since sunrise as a categorical variable in the regression model and used AIC as a measure of the support for this variable.

For analyses pertaining to individual variation, we report averages and their associated standard deviations (SDs); for analyses pertaining to variation due to time (time since sunrise, photoperiod) or weather (*T*_min_, wind speed, precipitation), we report the rate at which the mean total daily visits changed with changes in time or weather (slope) and the associated 95% confidence interval. The sample size for all analyses is *n* = 70 individuals unless otherwise noted.

## 3 RESULTS

Of 174 individuals captured and PIT‐tagged at our feeders, 70 birds returned and were detected by RFID data loggers (*n* = 25 individuals from pilot work conducted in winter 2015/2016, and *n* = 45 individuals tagged during winter 2016/2017), resulting in 315,745 visits across our 160‐day study period. Tagged birds took an average of 11 days (*SD* = 23.9, range = 0–118) to return to a feeder after their initial capture, and the average time it took birds to visit all feeders they ultimately used during the study was 37 days (*SD* = 53.4, range = 0–151). Feeders were highly variable in the total number of times they were visited by tagged birds during the study, with an average of 15,035 visits (*SD* = 9,678.2, range = 874–31,561). Similarly, the number of individual birds that used a particular feeder was also highly variable with an average of 11.5 birds (*SD* = 3.9, range = 3–20). The maximum distance we detected tagged birds moving between feeders was 1,850 m.

### 3.1 Temporal and individual variation in feeder use

**Figure 2:**
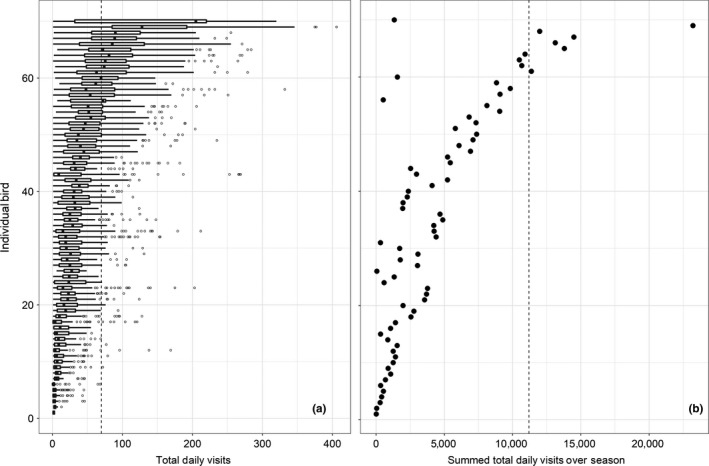
(a) Boxplots of total daily feeder visits for each Black‐capped Chickadee (*n* = 70) in our study from October 23, 2016–March 31, 2017 ranked from lowest number of total daily feeder visits to highest number of total daily feeder visits. The vertical dashed line represents the approximate number of visits required if an individual obtained all its daily energy from our feeders. (b) Summed total daily feeder visits for each chickadee. Vertical ordering of chickadees is the same as panel (a). The vertical dashed line represents the approximate number of seeds required if an individual received all its daily energy from feeders for the entire length of the study

Of the 160 days that feeders were available to chickadees, individuals visited feeders an average of 103 days (*SD* = 47.7, range = 2–160) which equated to 66% of the study period. Over the course of the study, the number of visits to any single feeder by an individual bird ranged from 1 to 15,512, and individuals averaged 4–9,831 visits per feeder (mean = 1,454, *SD* = 1,269). We found substantial variability in total daily visits among individual chickadees, with an average of 44 visits/day (*SD* = 46.3, range = 1–406 visits/day; Figure 2). Of note, the variation in total daily visits among birds was 70x greater than the variation within birds, indicating large, consistent differences in the extent that individual chickadees used feeders. Individual birds were also variable in the number of feeders they used. Of the 21 feeders that were available during our study, individual birds used an average of 4.5 feeders (*SD* = 2.2, range = 1–9 feeders).

**Figure 3:**
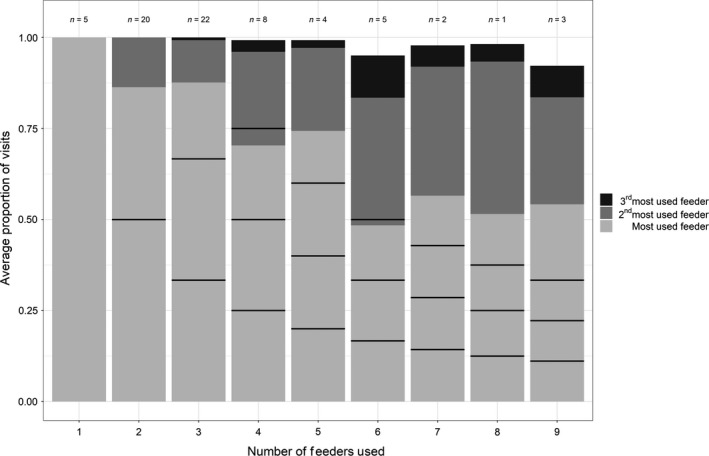
Average proportion of visits to the most‐visited, second‐most‐visited, and third‐most‐visited feeders for chickadees visiting multiple feeders during our study; sample sizes for each group are listed above each bar. Black horizontal lines within each bar indicate the expected proportion of visits if birds used all feeders equally. Because the proportions in each vertical bar are averages taken over birds, the sum of all proportions within each bar does not equal 100% for groups with 4–9 feeders used. The light gray portion of each bar indicates the largest observed proportion of feeder visits for each group

**Figure 4:**
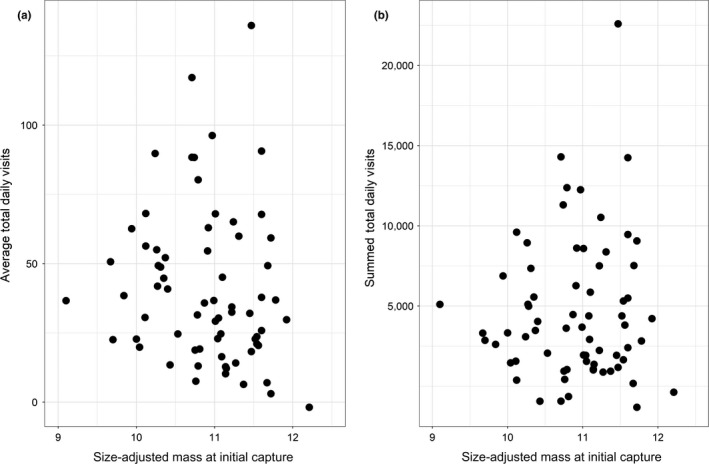
Relationship between size‐corrected body mass and (a) average total daily visits and (b) summed total daily visits of PIT‐tagged Black‐capped Chickadees

Approximately 93% of the tagged individuals that returned to feeders eventually visited the feeder at which they were captured, and 50 of our tagged birds (71%) visited only 2–4 feeders during the entire study period (Figure 3). No matter how many feeders were visited by individuals across the season, the average proportion of visits to the feeder visited most often was always greater than what would be expected if birds evenly distributed their visits across the feeders they used (Figure 3); thus, individuals appeared to favor some feeders over others. Although individuals varied markedly in body mass at initial capture (9.1–12.5 g), size‐adjusted body mass (i.e., with tarsus length fitted as a covariate) was unrelated to either average total daily visits (*F*_1,65_ = 1.22, *p* = 0.27, *R* = −0.14) or summed total daily visits (*F*_1,65_ = 0.13, *p* = 0.72, *R* = 0.05; Figure 4).

### 3.2 Relationship between feeder use and environmental variables

**Figure 5:**
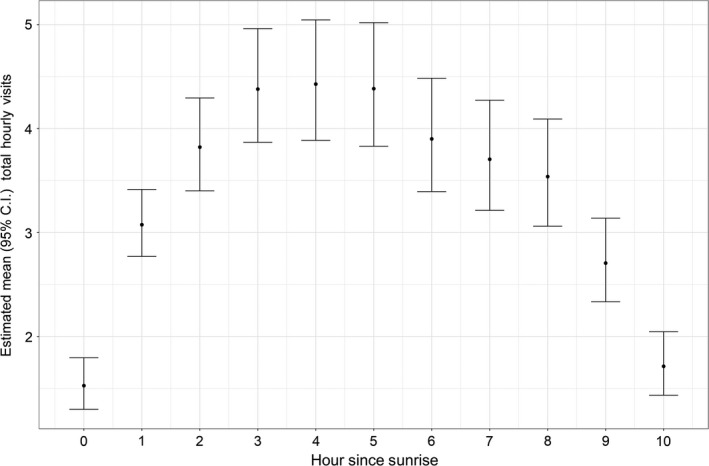
Estimated mean (± 95% CI) total hourly visits for Black‐capped Chickadees for each hour since sunrise during the shortest days of the year (total day length = 8.8–9.8 hr) with respect to photoperiod for the period November 9, 2016 through January 31, 2017

**Figure 6:**
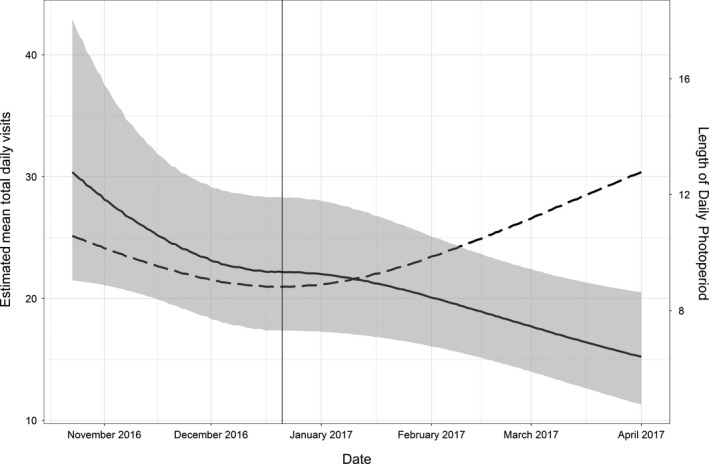
Estimated mean total daily feeder visits (solid line, left vertical axis) by PIT‐tagged Black‐capped Chickadees as a function of photoperiod based on a generalized linear mixed model. Solid vertical line denotes winter solstice. Dashed line is length of photoperiod in hours (right vertical axis). Prior to the solstice, feeder use rates decreased as photoperiod decreased, and feeder use continued to decrease after the solstice as photoperiod increased

We found strong support for an effect of the hour since sunrise on total hourly visits compared to the null model (∆AIC = 490.8). Total hourly visits rapidly increased after sunrise, peaked during 3–5 hr after sunrise, and then gradually decreased until sunset (Figure 5). We detected an effect of photoperiod on mean total daily visits such that they differed between the pre‐ and postsolstice periods (Table 1). In particular, estimated mean total daily visits decreased by 1.4 visits (95% CI: 1.0, 1.9) as photoperiod decreased from the start of our study to the winter solstice, and mean total daily visits decreased by 1.5 visits (95% CI: 1.1, 2.0) as photoperiod increased after the solstice until the end of our study (Figure 6). Thus, mean daily visits continued to decline over time during our study despite the reversal in photoperiod after the winter solstice. The estimated mean total daily visits prior to the solstice decreased by 18% (95% CI: −21, 2) for every 1 hr of decrease in photoperiod up until the winter solstice, after which estimated mean total daily visits decreased by 9% (95% CI: −16, 2) for every 1 hr of increase in photoperiod. In contrast to an effect of photoperiod, we found no support for an influence of *T*_min_, wind speed, or precipitation on total hourly visits, or for weather variables occurring in combination (Table 1); *T*_min_, wind speed, and precipitation were each estimated to change the estimated mean daily feeder visits by a factor of 1.0 (95% CI: [0.99, 1.00] for all 3 variables). Thus, we found no evidence that weather variables were linked to our measure of daily feeder use. During our study average daily *T*_min_ was 2.1°C, average daily wind speed was 8.7 km/hr, and average daily precipitation was 6.6 mm, all of which were similar to 30 years averages (i.e., 1.9°C, 7.4 km/hr, and 5.2 mm, respectively).

**Table 1:** Information Criterion statistics for generalized linear mixed models used in modeling feeder use by Black‐capped Chickadees (*Poecile atricapillus*) relative to environmental variables in Corvallis, Oregon, USA

Model	Explanatory Variables	K	Δ AIC	W	ER	AIC
1	Photoperiod + Solstice*Photoperiod	7	0	0.825	1.000	62456.5
	Photoperiod + Solstice*Photoperiod + Tmin + Wind Speed + Precipitation	10	3.7	0.130	6.360	62460.2
	Photoperiod	6	6.2	0.037	22.198	62462.7
	Null	5	9.1	0.009	94.632	62465.6
						
2	Null	5	0	0.470	1.000	62465.6
	Precipitation	6	0.7	0.331	1.419	62466.3
	Precipitation + Solstice*Precipitation	7	2.7	0.122	3.857	62468.3
	Precipitation + Solstice*Precipitation + Tmin + Wind Speed + Photoperiod	10	3.6	0.078	6.050	62469.2
3	Null	5	0	0.590	1.000	62465.6
	Tmin	6	1.9	0.228	2.586	62467.5
	Tmin + Solstice*Tmin + Wind Speed + Precipitation + Photoperiod	10	3.6	0.098	6.050	62469.2
	Tmin + Solstice*Tmin	7	3.9	0.084	7.029	62469.5
4	Null	5	0	0.523	1.000	62465.6
	Wind Speed	6	1.9	0.202	2.586	62467.5
	Wind Speed + Solstice*Wind Speed + Tmin + Precipitation + Photoperiod	10	2.2	0.174	3.004	62467.8
	Wind Speed + Solstice*Wind Speed	7	3.3	0.100	5.207	62468.9

Solstice is an indicator variable for the postsolstice period. Models are ordered according to weather variable and then by Akaike's Information Criterion (AIC). K denotes the number of parameters in the model; ΔAIC denotes the difference in AIC from the model with the lowest AIC for each environmental variable; W is the Akaike model weight; ER is the evidence ratio.

## 4 DISCUSSION

Our study found that chickadees exhibited extensive variation in their use of bird feeders across the nonbreeding season. It is well known that individual organisms typically vary in their individual behavior (Koolhaas et al., 1999; Sih et al., 2012), and therefore, behaviorally mediated differences are likely to underlie the patterns we observed in our study. For example, individuals that are bolder in their exploration of their environment are also more likely to engage in high‐risk activities (Koolhaas et al., 1999; Sih et al., 2012). Such individuals may frequent bird feeders more often, which could be considered risky environments given that feeders concentrate songbird feeding activities and can serve to attract predators (Lima, 1985; Dunn & Tessaglia, 1994; but see Roth, Vetter, & Lima, 2008). Additionally, individual variation in physiology, particularly energetic expenditure (Biro & Stamps, 2010; Careau & Garland, 2012; Careau et al., 2008), could also have underlain the substantial individual‐level variation in feeder use we detected. Thus, the pronounced individual differences in feeder use we observed may not be particularly surprising; however, the magnitude of the variation was rather unexpected, considering that some individual chickadees limited their use of feeders to a few visits each day, whereas others visited feeders several hundred times in a given day. Such stark differences in individual use of feeders are consistent with recent work on chickadees (Latimer et al., 2018) as well two closely related species, the Great Tit (*Parus major*) and Blue Tit (*Cyanistes caeruleus*; Crates et al., 2016). That this pattern was detected across three closely related species (Johansson et al., 2013) leads us to suspect that such variation might be typical of members of the Paridae, and that additional studies of more distantly related species using PIT‐tagged individuals and RFID‐equipped feeders will show this pattern of feeder use is prevalent in other groups. Given our study is one of the first to provide quantitative estimates of months‐long individual variation in use of feeders by wild songbirds during winter, future studies should build upon this work by using an experimental approach to evaluate putative mechanisms that may explain why individuals differed in their feeder use patterns, including the fitness consequences of such differences (see Crates et al., 2016).

Our analysis of the fat content of sunflower seeds indicated that an individual chickadee would require approximately 70 feeder visits each day to meet all of its daily energy requirements, assuming that chickadees acquire a single sunflower seed during each feeder visit (Ficken et al., 1990; Smith, 1991). This is a conservative estimate, so it was surprising that approximately 10% of our tagged birds averaged >70 feeder visits per day when evaluated across our entire study period, with a substantial number of individuals at times visiting feeders >200 times per day! In addition, we found that daily feeder use decreased across the winter season (i.e., both before and after the winter solstice), and was not solely driven by daylength. Both of these results are consistent with prior work that has found that chickadees cache food, and that caching behavior is most pronounced in the fall and decreases as winter progresses (Brodin, 2005; Sherry & Hoshooley, 2007; Smith, 1991). In a natural setting (i.e., without provisioning of supplemental food), this pattern may be due to a reduction in seeds produced during the growing season that typify the food items that are cached by chickadees (Brodin, 2005; Foote et al., 2010), or simply to an adequate number of caches prepared by birds in fall for future use. Our finding of what appears to be surplus feeder visits being maintained in a Mediterranean climate offers a notable contrast to the idea that energetically demanding environments result in stronger selection pressure for caching behavior (Croston et al., 2016; Pravosudov & Roth, 2013; Pravosudov, 2007). These findings also suggest that control over food‐caching may operate independently of local environmental conditions, and they indicate that more work on this topic is needed, especially around understanding the drivers of food‐caching during conditions when predictable, supplemental food is available (Pitera et al., 2018; Pravosudov, 2007).

Despite the pronounced individual variation in feeder use in our study, we did not detect a strong relationship between feeder visitation by chickadees and several weather‐related variables (i.e., minimum daily ambient temperature, wind speed, and precipitation) that has been found in previous studies within continental climates. Our findings regarding temperature are concordant with results from use of feeders by chickadees in Maine, USA (45°N), where feeder use was found to be unaffected by ambient temperature despite markedly colder conditions than those in our study area (i.e., lows to −18°C; Wilson, 2001). In contrast, chickadees in Wisconsin, USA (43°N; Latimer et al., 2018) and interior Alaska, USA (64°N; Kessel, 1976) increased their use of feeders with decreasing ambient temperatures. Given that ambient temperature is expected to have a strong influence on chickadee metabolic rate (Olson, Cooper, Swanson, Braun, & Williams, 2010) and, in turn, the extent to which supplemental food is used, some factor(s) other than temperature appears to be driving the previously identified relationships between feeder use and temperature. Characteristics of the areas where birds were sampled may be one such factor which could relate to alternative food resources, predator populations, or the quality of local habitats (Latimer et al., 2018).

Chickadees in our study followed a distinct pattern of daily feeder use during the shortest days of the year, increasing their use of feeders sharply after sunrise, with a plateau around mid‐day, followed by a gradual decrease until sunset. Theoretical models of optimal foraging posit that small birds should feed in a bimodal pattern, with heavy bouts of foraging in the morning and late in the day prior to roosting, with lower levels throughout the rest of the day (Bednekoff & Houston, 1994; McNamara et al., 1994). We predicted that would be the case with chickadees, but this was not supported by the daily pattern of feeder use by birds in our study system. One explanation is that weather conditions experienced by chickadees in our study area were insufficient to impose strong energetic challenges that would lead to predicted patterns of feeder visits. For example, work in a continental climate (Wisconsin, USA) found the lowest number of chickadees visiting feeders in the morning, and the highest feeding rates as sunset approached, with the latter possibly allowing birds to maximize energetic intake before nighttime roosting (Brittingham & Temple, 1992). Feeder use for chickadees gradually increased throughout the day in another continental climate (New York, USA), with an increase two hours prior to sunset and a sharp decrease in the hour before sunset (Bonter et al., 2013). These winter climates are markedly different from our system, where milder overnight temperatures would not require individuals to build up as extensive energy reserves for surviving the night. Thus, birds in our system may postpone body mass gain until later in the day to minimize predation risk while also reducing the energetic costs associated with carrying extra fat reserves for overnight survival (Farine & Lang, 2013; Lima, 1986; McNamara et al., 1994; Polo, Carrascal, & Metcalfe, 2007).

Shorter nights in lower latitude Mediterranean climates could increase the amount of time in a day available for foraging, such that birds may be able to distribute feeder use throughout the day differently than birds in higher latitude regions with continental climates. However, that did not appear to be the case in our study, as the latitude of our study site was generally comparable to several previous studies in continental climates and thus photoperiods should have been similar. This instead suggests that differences in energetic costs may be more important in driving patterns of feeder use. Regardless of the underlying mechanism(s), variation in the diurnal pattern of feeder use across regions suggests that small birds regulate their energy intake based on local conditions. In line with this, recent work in the closely related Mountain Chickadee (*P. gambeli*) found differences in visitation patterns to supplemental feeders whereby more visits were made early in the day when environmental conditions were harsher at higher elevation sites relative to lower elevation sites where visits were spread more evenly across daylight hours (Pitera et al., 2018). This pattern was thought to be due to a greater risk of starvation at sites with more severe winter weather such that individuals at those locations would benefit from foraging more frequently throughout the day with activity periods concentrated shortly after sunrise and prior to sunset (Roth & Pravosudov, 2009). Of note, the low elevation sites studied by Pitera et al. (2018) presented more challenging weather conditions that those in our study, yet the daily pattern of foraging was similar to what we observed. Thus, there may be a point at which feeding activity throughout the diurnal cycle shifts from an inverted U‐shaped distribution under less challenging conditions—as observed in this study—to a bimodal distribution under more challenging environmental conditions (Bednekoff & Houston, 1994; McNamara et al., 1994). If true, this helps to reconcile theoretical predictions with the observations we recorded in Oregon for chickadees relative to other parts of their range (New York; Bonter et al., 2013; Wisconsin: Brittingham & Temple, 1992), as well as the differences reported for Mountain Chickadee populations that experienced different environmental conditions during winter (Pitera et al., 2018).

We conducted our work in a setting where recreational bird feeders (i.e., feeders that were not part of our study) may have been used by our PIT‐tagged birds; however, we find that this possibility, if true, was unlikely to exert a strong influence on our results. First, all of our study feeders were placed on agricultural lands owned by Oregon State University, and therefore, few of our feeders were placed near residential homes; instead, the closest buildings were university‐owned buildings that had a low likelihood of harboring recreational feeders. Second, we found no relationship between the number of visits that birds made to our feeders and the distance to the nearest building. If recreational feeders were present at nearby buildings and reduced feeder visits by tagged birds in our study, we would have expected a relationship where the number of feeder visits would decline as distance to building decreased. That we found no evidence of such a relationship suggests that nearby buildings—assuming that they harbored recreational feeders—did not serve to influence birds visiting our feeders. Finally, all of our study feeders were located within 3.2 km of one another, and chickadees displaced from winter flocks can move up to 2.8 km in a single 24‐hr period (Odum, 1941). Moreover, winter flocks of chickadees can exhibit extensive overlap of wintering foraging areas (Desrochers & Hannon, 1989), so tagged chickadees in our study theoretically could have traversed much of our study area during the 5‐month period when we monitored feeder visits. If true, all tagged birds would have likely been exposed to the same pool of recreational feeders such that any influence of recreational feeders would be extended to all tagged birds. This means that visits to recreational feeders by tagged birds would not have introduced any systematic bias into our study but instead would have resulted in more conservative estimates of feeder use. Based on these points, we conclude that recreational feeders were unlikely to have a strong effect on our results, if they were indeed present and used by our tagged chickadees.

## 5 CONCLUSION

Our study in a Mediterranean climate found large variation among individuals in the extent of their use of supplemental bird feeders during winter, with limited influence of weather‐related variables that have been linked to feeder use patterns in continental regions experiencing colder winters. In particular, our study suggests that temperature may play a lesser role in driving the use of feeders by birds in warmer regions such as the Mediterranean climate in which we worked, perhaps because weather conditions were insufficient to impose strong energetic challenges to individuals. Nevertheless, this finding has implications for feeder use patterns in mild climates, as well as in continental climates where climate change is expected to result in winter warming (Zuckerberg et al., 2011). One important question that arises from our study is whether the variation in supplemental feeder visits was tied to the physiological health of individuals (Wilcoxen et al., 2015), which is a critical question for understanding why individuals varied in their use of feeders. For example, individuals that made a surplus of feeder visits may have been in relatively good body condition, and visited feeders at high rates to cache seeds for future use. Alternatively, chickadees with high use of feeders may have been in worse health relative to other individuals and therefore may have been more dependent on supplemental feeders to provide them with an easily accessible, constant, and predictable food source during challenging environmental conditions. Distinguishing between these alternatives cannot be accomplished via descriptive studies, so investigations that experimentally manipulate body condition and concurrently monitor feeder use are needed to evaluate the extent of feeder dependency across a range of environmental conditions.

## CONFLICT OF INTEREST

None declared.

## AUTHORS’ CONTRIBUTIONS

JLL, JWR, and LMG conceptualized and designed the study; JLL conducted field data collection; JLL and LMG conducted statistical analyses, with input from JWR; all authors were involved in writing and editing the manuscript.

## Data Availability

Data associated with this paper have been deposited in the Dryad Digital Repository https://doi.org/10.5061/dryad.9p47b7f.
