# Supplementary figures and images for: Individual variation and seasonality drive bird feeder use during winter in a Mediterranean climate
Source: Ecol Evol. 2019 Feb 14;9(5):2535–49. doi: 10.1002/ece3.4902 (PMC6405926; doi:10.1002/ece3.4902)

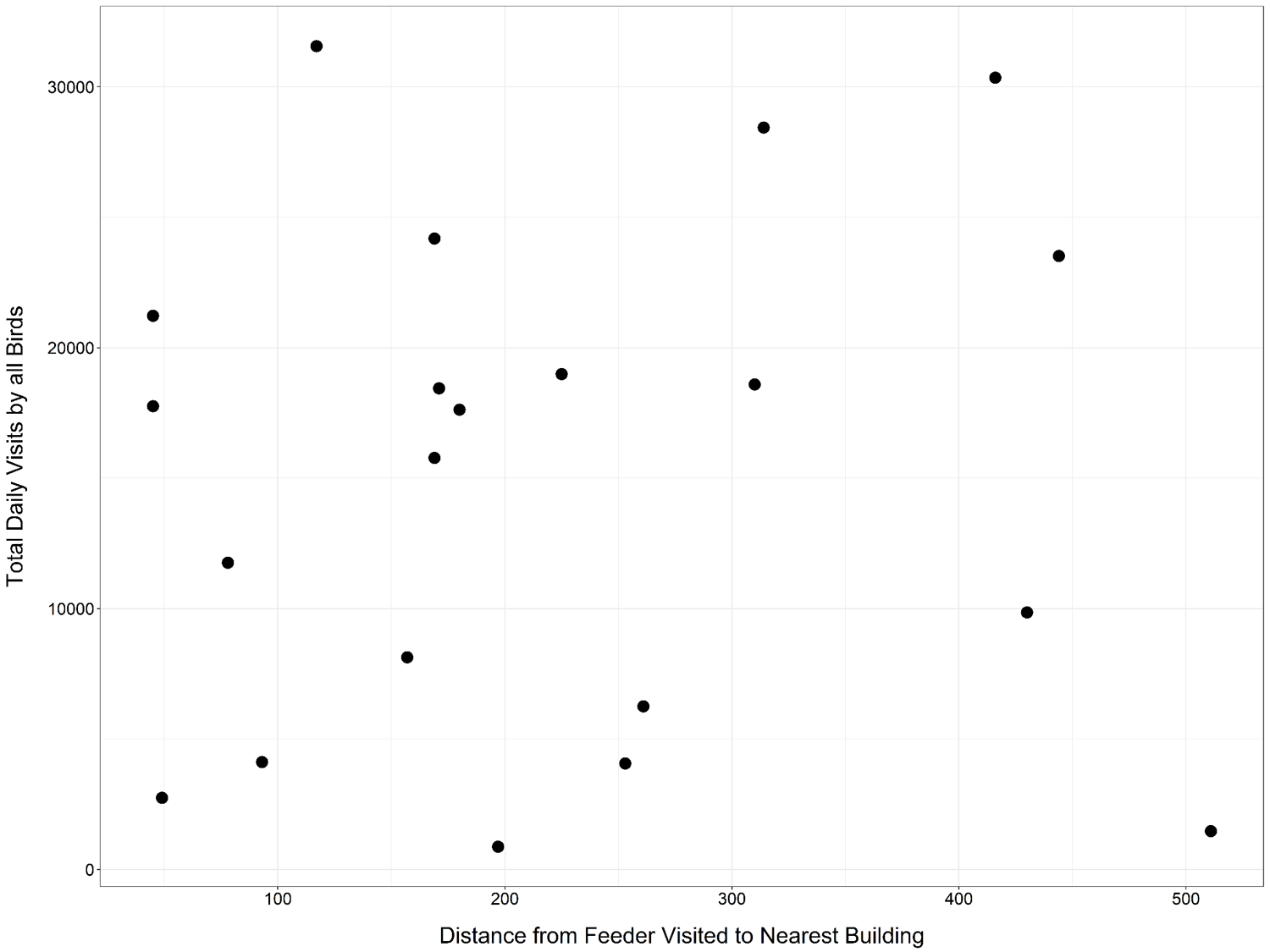

Supplement: Supplementary file 1 [file ECE3-9-2535-s001.png]

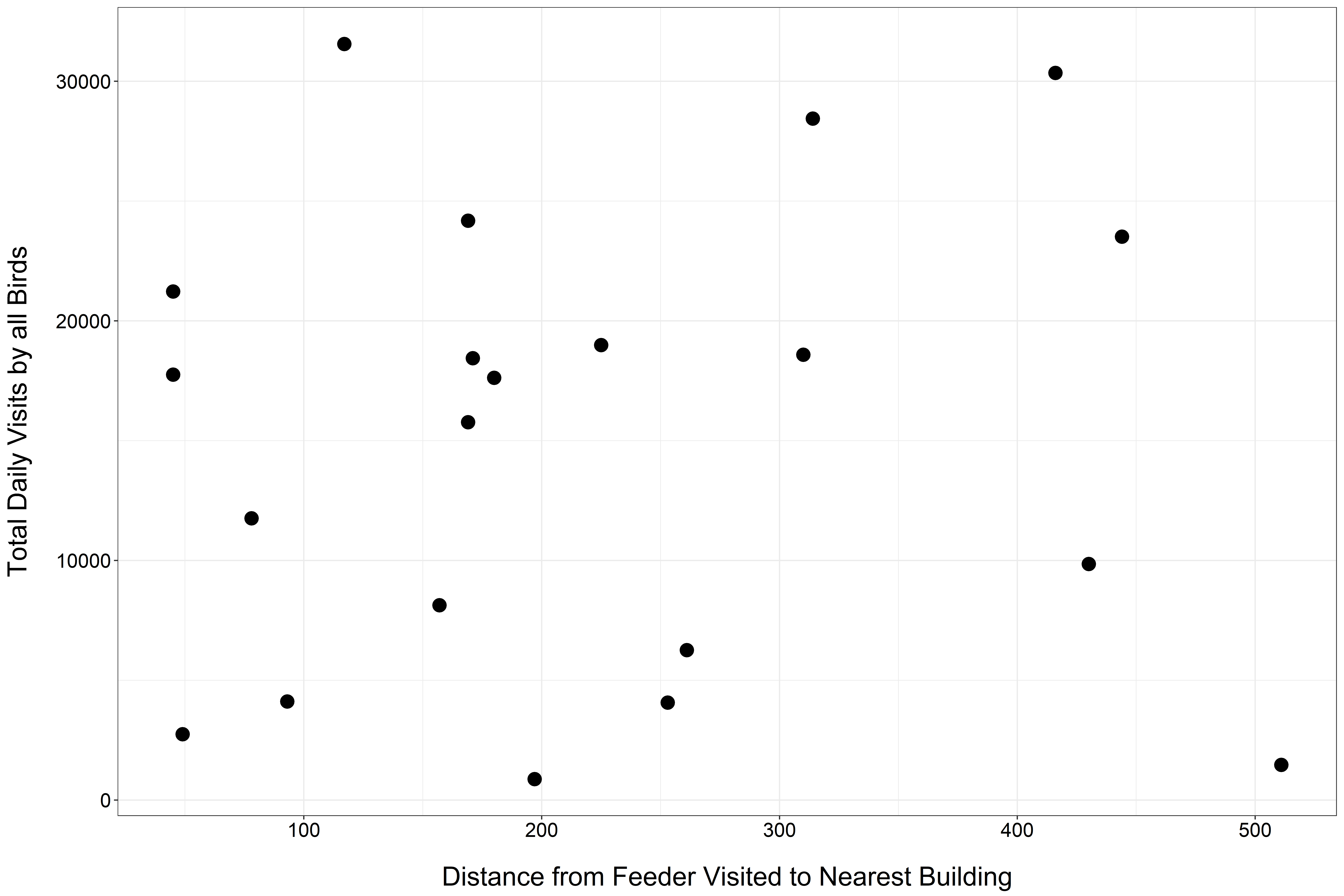

Supplement: Supplementary file 2 [file ECE3-9-2535-s002.tiff]
